# Similar effects on exercise performance following different respiratory muscle training programs in healthy young men

**DOI:** 10.1038/s41598-023-41580-w

**Published:** 2023-09-26

**Authors:** Dominic A. Notter, Samuel Verges, Andrea S. Renggli, Fernando G. Beltrami, Christina M. Spengler

**Affiliations:** 1 Exercise Physiology Lab, Institute of Human Movement Sciences and Sport, ETH Zurich, Gloriastrasse 37/39, 8092 Zurich, Switzerland; 2 Center for Integrative Human Physiology (ZIHP), University of Zurich, Zurich, Switzerland

**Keywords:** Respiration, Physiology

## Abstract

Both respiratory muscle endurance training (RMET) and inspiratory resistive training (IMT) seem to increase whole-body exercise performance, but direct comparisons between the two are scarce. We hypothesized that the similarity of RMET to exercise-induced ventilation would induce larger improvements compared to IMT. Twenty-six moderately-trained men performed either 4 weeks of RMET, IMT or SHAM training. Before and after the interventions, respiratory muscle endurance, 3-km running time-trial performance and leg muscle fatigue after intense constant-load cycling (assessed with femoral nerve magnetic stimulation) were measured. Both RMET (+ 59%) and IMT (+ 38%) increased respiratory muscle endurance (both *p* < 0.01 vs. SHAM) but only IMT increased inspiratory strength (+ 32%, *p* < 0.001 vs. SHAM). 3-km time improved showing a main effect of training (*p* = 0.026), however with no differences between groups. Leg fatigue after cycling was not attenuated with training (*p* = 0.088 for group-training interaction). All groups showed a significant (~ 0.3 l) increase in average tidal volume during cycling exercise combined with a concomitant reduction in respiratory exertion. While RMET and IMT improved specific aspects of respiratory muscles performance, no benefits beyond SHAM were seen during whole-body exercise. Changes in respiratory sensations might be a result of altered breathing pattern.

## Introduction

During whole-body exercise in healthy individuals, the respiratory system does not limit the capacity to reach maximal oxygen uptake^1^. During endurance events, on the other hand, the respiratory muscles are susceptible to fatigue, with negative consequences for exercise performance^2^. This is believed to be due to the respiratory muscle metaboreflex, whereby fatigue of respiratory muscles triggers vasoconstriction in the lower limbs, ultimately exacerbating lower limb fatigue by compromising limb blood flow and thereby exercise performance^3^.

Thus numerous studies have tested the ability of specific respiratory muscle training (RMT) to improve respiratory muscle endurance, strength, and exercise performance in healthy participants^4,5^. Two different forms of RMT regimes have mainly been employed: (i) respiratory muscle endurance training in form of volitional normocapnic hyperpnea (RMET^6–9^) and (ii) inspiratory muscle training using external resistances or threshold loads (IMT^8,10–12^). Although there is some overlap with regards to the training adaptations elicited, RMET—addressing both inspiratory and expiratory muscles—has more pronounced effects on respiratory muscle endurance whereas IMT is better suited for improving the strength of the inspiratory muscles^13^. It has been hypothesized that RMET could better translate into ergogenic effects during whole-body exercise^13^, as the ventilatory demand placed upon the respiratory muscles during RMET is higher than that of high-intensity whole-body exercise. Furthermore, RMET but not IMT was shown to reduce blood lactate concentrations during hyperpnea^13^, and RMET loads the inspiratory as well as the expiratory muscles, both of which can contribute to an increase in sympathetic discharge when fatiguing^14^.

The only investigation to date that tested both training protocols found IMT to be superior to RMET in terms of ergogenic effects, even though the latter showed better results during a specific respiratory muscle endurance test^9^. This experiment, however, was performed during swimming, an activity where the load placed on the inspiratory muscles is increased compared to land-based exercise. Even within land-based exercise modalities, the activation and function of the respiratory muscles will also differ, meaning for example that ergogenic effects seen after RMT in cycling might not translate into running, where the respiratory muscles also play a role in core stabilization^15–17^, and vice-versa. The impossibility to generalize results from one activity to the other further compounds the issue of lack of direct comparisons, even if indirect evidence suggests that respiratory muscle fatigue reaches similar levels after cycling and running trials, pointing towards the potential for RMT to enhance performance in both modalities^18^.

The precise physiological mechanisms by which either version of RMT might improve whole-body exercise performance remain unclear. Although evidence from acute interventions suggest a role for the respiratory muscle metaboreflex in regulating performance, whereby loading or unloading inspiratory muscles has opposing effects on leg blood flow and fatigue^2,19–22^, evidence from training studies do not support this hypothesis^23^. Likewise, evidence from a meta-analysis suggests that the ergogenic effects of RMT are greater during longer events^4^, which is at odds with the indication that diaphragmatic fatigue (and thus the respiratory muscle metaboreflex) is more common when intensity exceeds 85% of maximal oxygen consumption^24^. Finally, while RMT does not seem to influence cardiovascular responses during exercise^25,26^, some studies reported a decrease in the perception of breathlessness and/or respiratory effort^7,11,12,27^, which points towards perceptual changes having an influence on performance, perhaps independently of the absolute levels of muscle fatigue developed.

We therefore aimed to test whether the potentially different effects of the two RMT regimes would translate into different changes in running and cycling performance, and also in leg muscle fatigability. We hypothesized that both RMET and IMT would improve respiratory muscle endurance, running time-trial performance and cycling endurance at constant ventilation and that leg muscle fatigability would be reduced in both training groups compared with SHAM training, but that larger effects would be seen following RMET.

## Methods

### Ethical approval

All participants gave their written informed consent prior to enrollment. The protocol was approved by the ethics committee of ETH Zurich (2003-0427) and performed according to the Declaration of Helsinki, except for registration in a public database.

### Participants

Sample size determination was performed using G*Power. Using a repeated-measures ANOVA with within and between factor interactions, a sample size of n = 24 for three groups undergoing two assessments each was determined, with an estimated power of 80% to detect an effect size (according to Cohen) of 0.7 at an alpha error probability of 0.05. While this is considered a large effect size, it is in line with research in the field^28^ and the magnitude of gains seen after similar interventions in running^6^.

Twenty-six active, non-smoking, male participants with normal lung function were randomly assigned to one of three groups: (1) RMET-group (n = 9; age: 28 ± 4 y, height: 180 ± 5 cm, mass: 72 ± 4 kg, peak oxygen consumption (${\dot{\text{V}}}$O_2peak_): 55.6 ± 7.9 ml∙min^−1 ^∙kg^−1^), (2) IMT-group (n = 9; age: 26 ± 7 y, height: 183 ± 6 cm, mass: 76 ± 6 kg, ${\dot{\text{V}}}$O_2peak_: 53.6 ± 7.6 ml∙min^−1 ^∙kg^−1^) or (3) a SHAM-group (n = 8; age: 27 ± 7 y, height: 181 ± 6 cm, mass: 72 ± 7 kg, ${\dot{\text{V}}}$O_2peak_: 53.2 ± 6.6 ml∙min^−1 ^∙kg^−1^).

### General design

The study was single blinded, i.e. the experimenters performing the tests did not know which group the participants belonged to. On the first visit, participants were thoroughly familiarized with the testing procedures, including performing maximal voluntary contractions, performing magnetic stimulations of the quadriceps and a complete assessment of lung function and respiratory muscle strength. Participants then visited the laboratory on five occasions prior to and following the training period, each two visits separated by at least 48 h. The following assessments were performed: lung function (day 1), an incremental exercise test on a cycle ergometer (day 2), a constant load time to exhaustion cycling test at 85% of maximal power output (W_max_) from the incremental test (day 3), a 3-km running time-trial (day 4) and finally, a respiratory muscle endurance test (day 5). This sequence was repeated after the training program (see below).

Participants were requested to keep their individual training routine constant for at least 2 weeks prior to, and throughout the course of the study. All training details, including the respiratory muscle training sessions, were entered into a diary (modality, duration, intensity and rate of perceived exertion) that was regularly checked by the experimenters to ensure participants’ compliance. Participants were instructed to not perform any strenuous physical exercise 2 days before the test sessions, and no physical exercise the day before the tests. Participants drank no caffeinated beverages on the day of the tests and they had their last meal at least 2 h before the tests. On each exercise test day, participants were given 0.5 L of an isotonic beverage (Isostar Long Energy; Wander, Bern, Switzerland) 2 and 4 h prior to the experiment (or the evening before, if the test took place within 2–4 h of waking). Assessments before and after the training period were scheduled for the same time of day ± 2 h.

#### Lung function

Lung function (Oxycon Alpha Plus, Höchberg, Germany) and maximal respiratory pressure (Validyne MP45, Northridge, California, USA) were assessed according to standard recommendations of the American Thoracic Society^29^. Following the assessments, participants were familiarized with the SpiroTiger device (Idiag, Fehraltorf, Switzerland) later to be used for the respiratory muscle endurance test and RMET (for details see below).

#### Incremental exercise test

Participants started cycling (Ergometrics 800S; Ergoline, Bitz, Germany) at 100 W for 2 min with subsequent increases of 30 W every 2 min until exhaustion. W_max_ was calculated as the workload of the last completed stage plus the number of seconds during the last (uncompleted) stage times 0.25 (thus equating 30 W for every 120 s cycled, the workload and duration of each stage of the test)^23^. Participants could choose a cycling cadence between 70 and 100 rpm within the first minutes of the test and once established it was kept within ± 3 rpm. The test ended when participants stopped or when the cadence could no longer be sustained within the designated range. Ventilation and gas exchange (Oxycon Alpha plus) were recorded breath-by-breath and heart rate (HR) was recorded continuously (Polar Vantage, Polar Electro, Kempele, Finland). The metabolic cart was calibrated for volume and gas fractions according to the manufacturer’s recommendation before and after each trial, to ensure that no technical issue or drift was present during the tests. 20 µl of capillary blood were drawn from an earlobe to analyze for blood lactate concentration ([La], Biosen 5040, EKF Diagnostic, Barleben, Germany). Finally, the participants were familiarized again with the use of the SpiroTiger device.

#### Cycling endurance test

During the cycling test performed prior to the training period participants cycled to exhaustion at 85% W_max_ following a brief warm-up (2 min at 40 and 2 min at 60% W_max_), an intensity which is considered sufficient to induce diaphragmatic fatigue following cycling^24,30,31^. Ventilation and gas exchange were recorded breath-by-breath and HR was recorded continuously. During the test, the participants were instructed to increase their pulmonary ventilation (${\dot{\text{V}}}$_E_) to 110% of the ${\dot{\text{V}}}$_E_ achieved in the last minute of the incremental test. Visual feedback was provided to assist participants in reaching the target, which could be done with any combination of tidal volume (V_T_) and breathing frequency (f_B_). ${\dot{\text{V}}}$_E_ was coached in an attempt to ensure similar levels between the assessments performed before and after the training period, so that any differences seen in quadriceps fatigue would be caused by differences in respiratory muscle fatigue resistance and not respiratory muscle work. Upon exhaustion, participants had a 5 min break when the assessment of quadriceps contractility was performed, and then resumed cycling under the same conditions, again to exhaustion. Blood samples were taken from the earlobe at rest, every 2 min during cycling and directly after both cycling bouts. After this, the participants did the last familiarization trial with the SpiroTiger.

The post-training cycling test mimicked the pre-training test, however the first ride was interrupted at the same moment as the pre-training assessment (termed isotime). As in the pre-training test, a 5-min break for the assessment of quadriceps contractility took place after which participants resumed cycling, this time until exhaustion. The cycling endurance test was designed this way so that quadriceps contractility could be assessed after similar work had been performed (by both the legs and respiratory muscles, from the first ride) and that an objective measure of endurance could be obtained (total cycling time, combining the two rides and leg fatigue at the end of the test).

#### 3-km time-trial

The time-trial was performed indoor in a sports building in order to have similar environmental conditions^32^ before and after the training phase. The time-trial consisted of 23 laps (130.5 m each) run in a figure of eight, which participants were instructed to complete in the shortest time possible. HR was recorded continuously during the trial whereas blood samples were taken from an earlobe to measure [La] before and after the standardized warm-up as well as immediately after the end of the time-trial.

#### Respiratory endurance test

Participants performed the respiratory endurance test (normocapnic hyperpnea) to exhaustion using the SpiroTiger device, as previously published^33^. Participants were required to maintain the target ${\dot{\text{V}}}$_E_ (70% of maximal voluntary ventilation measured over 15 s; MVV_15_) while holding V_T_ and f_B_ constant at a duty cycle of 0.5. The test ended either when participants reached volitional exhaustion, when 40 min were reached, or when the experimenter stopped the test after the 3^rd^ encouragement of the subject to either increase V_T_ or f_B_ to maintain the target ${\dot{\text{V}}}$_E_. The assessments performed before and after the training period were based on the pre-training MVV_15_.

#### Training

The RMET-group completed 20 training sessions of 30-min duration with 1 day of rest between 2 days of consecutive training using the SpiroTiger device^34^. ${\dot{\text{V}}}$_E_ of the first training session was set at 60% of the individual MVV_15_, with V_T_ set at 50–60% of forced vital capacity and f_B_ adjusted accordingly. If after 25 min of training participants felt that they would not be exhausted after 30 min of training, they were instructed to increase f_B_ by 5 breaths∙min^−1^ for the last 5 min of the training. The next training session then started with an increased f_B_ of 2 breaths﻿∙min^−1^ relative to the start of the previous session. If subjects could not increase f_B_ after 25 min of training, the next training session started with a f_B_ increased by 1 breath﻿∙min^−1^. If subjects felt after 25 min of training that they were not able to continue for another 5 min at the same target, they were allowed to decrease f_B_ by 5 breaths﻿∙min^−1^ and started the next training session with identical settings to the previous training.

The IMT-group trained twice a day every day for thirty days with an inspiratory resistance-training device (DeVilbiss RT-Trainer; Hounslow, UK), as previously described^13^. Before each training session, participants performed three maximal inspiratory maneuvers from residual volume to total lung capacity against the internal resistance (i.e. inspiring through a small hole). The breath that achieved the highest area under the curve (AuC) was taken as the reference breath for the training session. This constant re-evaluation of maximal force ensured training progression. The software calculated a curve referring to 80% of this pressure at any time point. This curve had to be reproduced 30 times by the participants. The duration of each breath was about 20 s. After each breath, a 10-s break with spontaneous breathing followed. Each breath needed to have an AuC of at least 95% of the target curve or it was discarded by the system and the subject had to repeat it. The AuC was recorded as an index of the work performed. This resulted in an average training load of 60% ± 4% MIP.

The SHAM-group trained twice a day every day using an incentive spirometer (Voldyne 5000, Sherwood Medical, St Louise USA): slow, complete exhalations were followed by slow inspirations up to 70% of their vital capacity, producing a constant flow controlled by the feedback of the device. This procedure was repeated every 30 s, paced by a metronome, for 15 min, resulting in 30 inspirations per session. Participants were told they were training to open all alveoli and to improve respiratory coordination resulting in better gas exchange and thus better performance.

﻿To verify compliance, HR was recorded during each session using a heart rate monitor (Polar Vantage, Polar Electro, Kempele, Finland). Training data of RMET and IMT were recorded by the training system and checked during each laboratory visit. In addition the settings of every training were recorded by the subjects in a diary. Respiratory trainings were performed at home, except for every fifth training session, which was supervised in the laboratory to ensure correct performance. For the RMET-group, the SpiroTiger was connected to the metabolic cart to ensure normocapnia was maintained and if participants felt uncomfortable with the setting of V_T_ and f_B_ at the ${\dot{\text{V}}}$_E_ they had reached, V_T_ was changed and f_B_ was adjusted accordingly.

### Perceptual responses

Breathlessness and respiratory exertion were each assessed by means of a visual analogue scale ranging from 0 (none) to 10 (maximal). To ensure a proper understanding of the terms, participants were extensively questioned about their prior experience with different respiratory sensations^35,36^. Thereafter, a definition was given for respiratory exertion (how hard it is to breathe) and clearly distinguished from breathlessness (the sensation of not getting enough air). Leg exertion was defined as “how hard it is to pedal”. Perceptual responses were assessed before, every 2 min and immediately after the cycling tests.

#### Quadriceps contractility

The presence of leg muscle fatigue was objectively determined by assessing quadriceps muscle contractility during magnetic femoral nerve stimulation (isometric knee extension force, Q_tw_). Measurements were performed before and after each of the two parts of the cycling endurance test. The participant started sitting in a custom-made chair and the back rest was slowly reclined until the correct (supine) position was adopted to ensure optimal placement of the stimulator, while the knee was kept flexed in a 90° position with the leg passively stabilized to prevent lateral motion.

A nonelastic ankle strap was attached to the force transducer (strain gauge LC4102-K060, A&D CO, Tokyo, Japan). The femoral nerve was stimulated with a 43-mm figure-of-eight coil powered by a magnetic stimulator, Magstim 200 (Magstim, Whitland, England), at the position which resulted in the highest Q_tw_. Just before the first stimulation, participants performed three isometric maximum voluntary contractions (MVC) for 5 s each to avoid the confounding effect of potentiation. Then, participants performed one 5-s MVC after a group of three stimulations. A minimum of nine stimulations was performed at 100% of the stimulator output. For analysis, the first three Q_tw_ were discarded, and Q_tw_ #4 to #9 were averaged^37^. Stimulation supramaximality was confirmed by the attainment of a plateau in quadriceps force at submaximal intensities of the stimulator. This procedure was performed before and after the cycling trials.

### Data analysis

Data are presented as mean ± SD. The effects of training on the different variables were compared using 2-way ANOVA (factors group and training), with Tukey’s posthoc when required. When data failed normality tests, log transformation was successfully used. Data from Q_tw_ was expressed as a percentage of baseline values, and a 2-way ANOVA was run between the different groups for the Q_tw_ values after the first exhaustion/isotime, as these had comparable workloads (factors group and training). The Q_tw_ at the end of the second exhaustion trial (open ended both before and after training) were compared on a separate 2-way ANOVA. Effect sizes for within group changes following training are presented as Cohen Dz (average difference / standard deviation of differences), along with 95% CI for differences. All calculations were performed using Prism 9.0 (Graphpad, La Jolla, CA). *P* < 0.05 was considered as statistically significant.

## Results

Training compliance of 100% was achieved in all three groups, as verified through personal interviews, the training logs and the HR files. The level of personal physical training was held constant throughout the study period when compared to the 2 weeks preceding the start of the training period (based on hours of physical activity per week).

### Lung function and respiratory muscle performance

The results from the respiratory endurance test and lung function in this cohort have been fully explored elsewhere^13^ and are briefly presented here for contextualization. Lung function data as well as MIP and breathing duration during the respiratory endurance test before and after training are shown in Table 1. There was a significant main effect of training for MVV_15_ (*p* = 0.002). MIP improved with training only for the IMT group (*p* = 0.014 vs. SHAM at post-training). Both RMET and IMT training regimes significantly increased breathing endurance during the respiratory endurance test (Table 1), with no difference in test results (test duration capped at 40 min).

**Table 1:** Lung function and respiratory muscle performances pre and post respiratory muscle endurance training (RMET), inspiratory muscle resistive training (IMT) and SHAM training.

	RMET	IMT	SHAM
FVC (l)			
Pre	5.8 (0.6)	6.3 (0.5)	5.6 (0.5)
Post	5.9 (0.8)	6.4 (0.9)	5.6 (0.6)
FEV_1_ (l)			
Pre	4.7 (0.3)	5.1 (0.5)	4.7 (0.6)
Post	4.8 (0.4)	5.1 (0.8)	4.7 (0.6)
PEF (l∙s^−1^)			
Pre	11.0 (2.1)	11.1 (1.5)	10.8 (1.4)
Post	11.2 (2.3)	11.2 (1.6)	10.5 (1.8)
MVV_15_ (l∙min^−1^)			
Pre	191.9 (25.7)	196.4 (19.3)	192.0 (22.9)
Post	205.5 (29.4) ^†^	205.2 (31.2) ^†^	202.2 (20.3) ^†^
V_T_ MVV_15_ (l∙min^−1^)			
Pre	2.0 (0.3)	1.9 (0.4)	2.1 (0.3)
Post	2.3 (0.3) ^†^	2.3 (0.5) ^†^	2.5 (0.6) ^†^
MIP (cm H_2_O)			
Pre	− 128 (33)	− 121 (28)	− 109 (25)
Post	− 124 (45)	− 160 (28)*^‡^	− 111 (30)
Breathing duration (min)			
Pre	25.2 (7.2)	26.2 (5.2)	26.6 (3.1)
Post	40.0 (0.0)*^‡^	36.2 (6.4)*^‡^	30.1 (7.0)

Values are Mean (SD); FVC, forced vital capacity; FEV_1_, forced expiratory volume in 1 s; PEF, peak expiratory flow; MVV_15_, maximum voluntary ventilation over 15 s; V_T_, tidal volume; MIP, maximal inspiratory pressure; Breathing Duration, duration of the respiratory endurance test. ^†^main effect for time (*p* < 0.05); * different from pre (*p* < 0.001); ^‡^different from SHAM at same time-point (*p* < 0.05).

### Incremental cycling test

**Figure 1:**
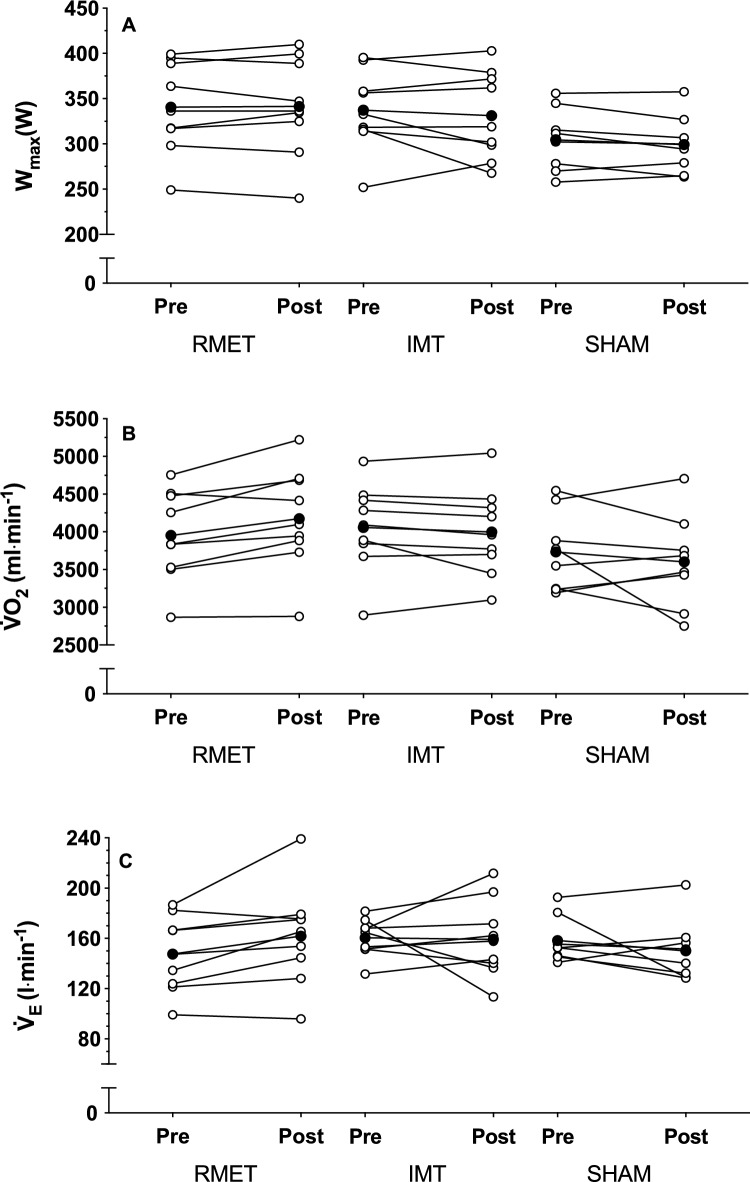
Maximal workload (**A**), peak oxygen uptake (**B**) and peak pulmonary ventilation (**C**) during an incremental cycling test performed before (Pre) and after (Post) respiratory muscle endurance training (RMET), inspiratory muscle resistive training (IMT) and SHAM training, given for individual participants (open circles) and as a group mean (closed circles).

There were no differences between groups in W_max_ (Fig. 1A, group-training interaction *p* = 0.65)_,_ ${\dot{\text{V}}}$O_2peak_ (Fig. 1B, group-training interaction *p* = 0.044) or the ${\dot{\text{V}}}$_E_ (Fig. 1C, group-training interaction *p* = 0.158) achieved at the end of the incremental test before or after training. There was a reduction in breathlessness at the end of the incremental test (from 3.8 ± 1.3 to 1.2 ± 0.5 points, all groups pooled, main effect of training *p* < 0.001) and an increase in leg exertion at the end of test (from 8.8 ± 0.5 to 9.6 ± 0.2 points, all groups pooled, main effect of training *p* = 0.002). V_T_ at the end of the incremental test was higher following the training period (from 3.1 ± 0.2 to 3.3 ± 0.2 l, all groups pooled, main effect of training *p* = 0.009).

### Constant-load cycling endurance test

Duration of the endurance cycling test did not show changes with training (group-training interaction effect *p* = 0.645). Physiological and perceptual responses during tests are shown in Table 2. Similar to the incremental test, average respiratory exertion throughout the test was lower (from 5.5 ± 1.0 to 4.5 ± 1.1 points, all groups pooled, main effect of training *p* = 0.010) and average V_T_ was higher (from 3.0 ± 0.2 L to 3.3 ± 0.2 L, all groups pooled, *p* < 0.001). Neither the absolute nor the relative changes in V_T_ and respiratory exertion correlated with each other (linear regressions for both r^2^ < 0.04). No group-training interactions were detected for leg exertion (*p* = 0.918) or HR (*p* = 0.329). At the end of the first cycling bout, however, leg exertion was ~ 11% lower (all groups pooled, main effect of training *p* = 0.022), while no differences were seen for respiratory exertion and breathlessness, and neither for HR nor blood lactate concentration (Table 2).

**Table 2:** Physiological and perceptual responses during cycling endurance test pre and post respiratory muscle endurance training (RMET), inspiratory muscle resistive training (IMT) and SHAM training.

	RMET	IMT	SHAM
Mean values			
${\dot{\text{V}}}$_E_ (l∙min^−1^)			
Pre	139.0 (28.7)	148.9 (23.4)	140.0 (22.5)
Post	138.7 (30.4)^†^	142.6 (25.0)^†^	135.3 (24.5)^†^
V_T_ (l)			
Pre	3.0 (0.5)	3.1 (0.8)	2.8 (0.7)
Post	3.4 (0.5)^†††^	3.3 (1.0)^†††^	3.1 (0.7)^†††^
f_B_ (breaths min^−1^)			
Pre	47 (8)	51 (17)	54 (17)
Post	41 (9)^†††^	48 (18)^†††^	46 (14)^†††^
${\dot{\text{V}}}$O_2_ (ml∙min^−1^)			
Pre	3520 (622)	3538 (577)	3290 (509)
Post	3654 (543)^†^	3558 (678)^†^	3351 (497)^†^
${\dot{\text{V}}}$CO_2_ (ml∙min^−1^)			
Pre	3481 (590)	3688 (507)	3381 (511)
Post	3668 (565)^†^	3660 (672)*^†^	3415 (466)^†^
P_ET_CO_2_ (mmHg)			
Pre	30.4 (2.2)	30.3 (4.6)	28.8 (2.9)
Post	31.8 (2.3)^†††^	31.5 (5.0)^†††^	30.4 (2.9)^†††^
HR (bpm)			
Pre	167 (10)	167 (12)	172 (9)
Post	169 (10)	166 (12)	170 (9)
Blood Lactate Concentration (mmol∙l^−1^)			
Pre	8.9 (1.9)	10.5 (1.5)	9.9 (1.8)
Post	9.0 (2.3)	9.7 (1.9)	9.8 (1.8)
Breathlessness (points)			
Pre	1.4 (2.4)	0.5 (0.6)	2.0 (1.9)
Post	0.4 (0.7)	0.6 (0.9)	1.9 (2.4)
Resp. Exertion (points)			
Pre	4.9 (2.0)	5.0 (2.3)	6.7 (1.8)
Post	3.7 (1.6) ^†^	4.1 (2.2) ^†^	5.7 (2.0) ^†^
Leg Exertion (points)			
Pre	5.9 (1.2)	5.9 (2.0)	6.2 (2.1)
Post	5.8 (1.7)	5.7 (2.4)	6.2 (1.0)
End Part 1 (isotime)			
HR (bpm)			
Pre	178 (9)	178 (12)	183 (9)
Post	179 (9)	176 (12)	181 (9)
Blood Lactate Concentration (mmol∙l^−1^)			
Pre	11.0 (2.6)	12.8 (2.3)	12.0 (2.1)
Post	10.9 (2.8)	12.0 (2.4)	11.9 (2.2)
Breathlessness (points)			
Pre	2.5 (3.8)	1.7 (3.0)	5.1 (3.8)
Post	1.1 (1.9)	1.1 (1.7)	3.0 (3.5)
Respiratory Exertion (points)			
Pre	8.1 (2.0)	7.7 (2.8)	9.0 (1.6)
Post	6.6 (2.4)^†^	6.2 (3.2)^†^	8.4 (2.5)^†^
Leg Exertion (points)			
Pre	9.6 (0.6)	8.6 (1.7)	8.6 (1.7)
Post	8.3 (1.3)^†^	8.1 (2.2)^†^	7.6 (2.3)^†^

As indicated, data presented are either the mean values of the equal time cycled during the pre- and post- assessments or the values collected at the end of the first cycling bout (isotime). Values are mean (SD). ${\dot{\text{V}}}$_E_, pulmonary ventilation; V_T_, tidal volume; f_B_, breathing frequency; ${\dot{\text{V}}}$O_2_, oxygen uptake; ${\dot{\text{V}}}$CO_2_, carbon dioxide output; P_ET_CO_2_, pressure of end tidal CO_2_; HR, heart rate. ^†^ main effect for time (^†^*p* < 0.05, ^††^*p* < 0.01, ^†††^*p* < 0.001); *different from before training (*p* < 0.05).

The within individual, between days coefficient of variation for baseline Q_tw_ was 12.4%. There were no differences in baseline twitches between the experimental visits (45.1 ± 10.5 Nm before training vs. 44.9 ± 11.1 Nm after training, all groups pooled, t-test *p* = 0.867). One participant from the RMET group and three from both the IMT and SHAM group did not reach isotime during the post-training trial, and their data was not included in this analysis.

**Figure 2:**
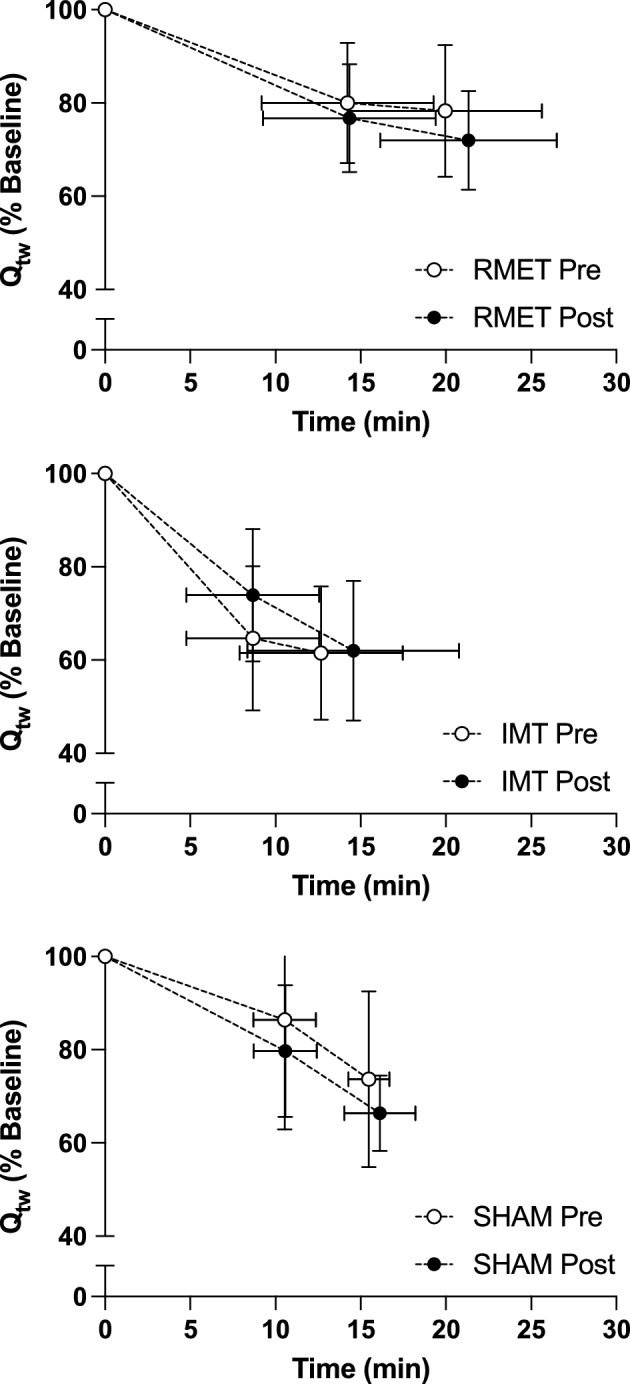
Mean ± SD for quadriceps twitch force (Q_tw_) reduction at isotime and at the end of the cycling endurance test before (open symbols, Pre) and after (closed symbols, Post) respiratory muscle endurance training (RMET, top), inspiratory muscle resistive training (IMT, middle), or SHAM training (bottom).

**Figure 3:**
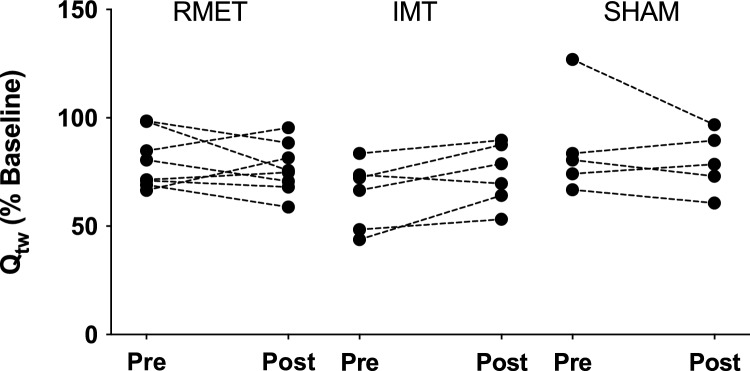
Individual values for quadriceps twitches (Q_tw_, as percentage of baseline values) measured after an equal workload of cycling before (Pre) and after (Post) a 4-week period of either respiratory muscle endurance training (RMET), inspiratory muscle training (IMT) or SHAM training.

Q_tw_ decreased nearly 30% from baseline levels following the first exhaustion/isotime (Fig. 2). Individual values for the drop in Qtw after the first exhaustion/isotime are shown in Fig. 3. No group-training interaction was noted for Q_tw_ after the first exhaustion/isotime (*p* = 0.086). Within groups, no changes were seen as an effect of training after either RMET (*p* = 0.827), IMT (*p* = 0.221) or SHAM (*p* = 0.543). Similarly, no group-training interactions were seen for Q_tw_ at the second exhaustion point (*p* = 0.561).

### 3-km running time-trial

**Figure 4:**
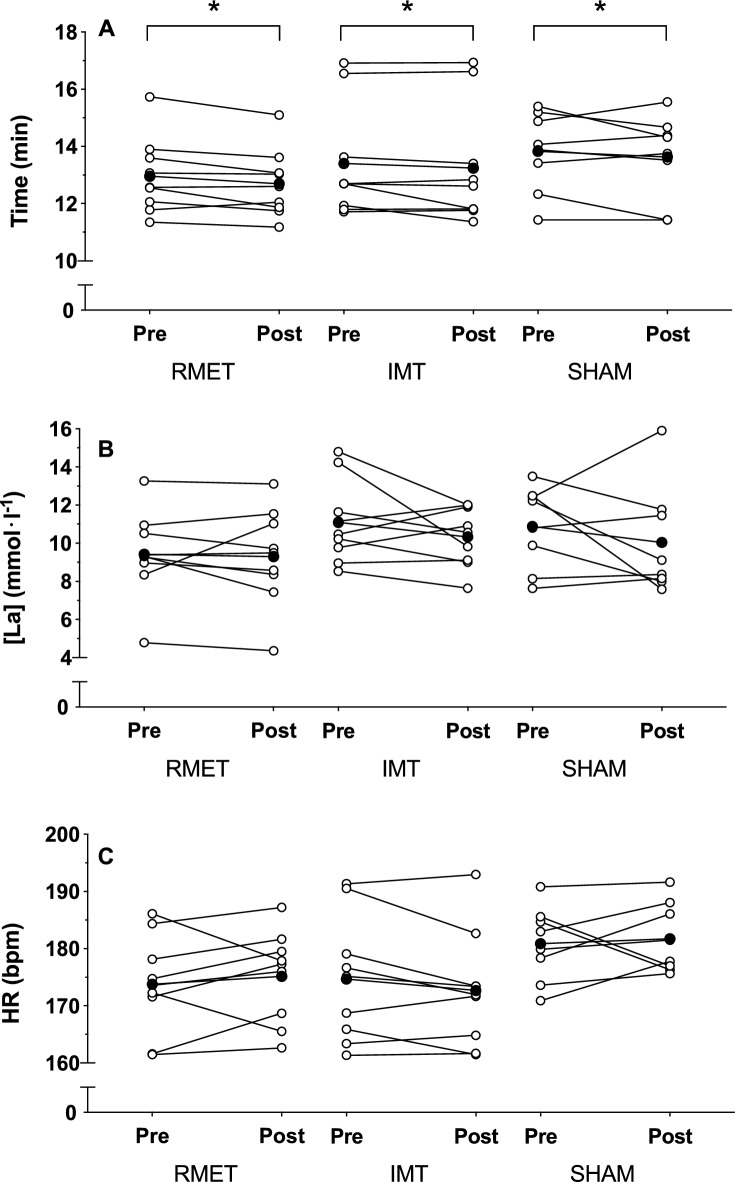
3-km running duration (**A**), blood lactate concentration at the end of the test ([La], **B**) and average heart rate (HR, **C**) before (Pre) and after (Post) respiratory muscle endurance training (RMET), inspiratory muscle resistive training (IMT) or SHAM training, given for individual participants (open circles) and as a group mean (closed circles). *main effect of training (pre vs. post) at *p* = 0.026.

**Figure 5:**
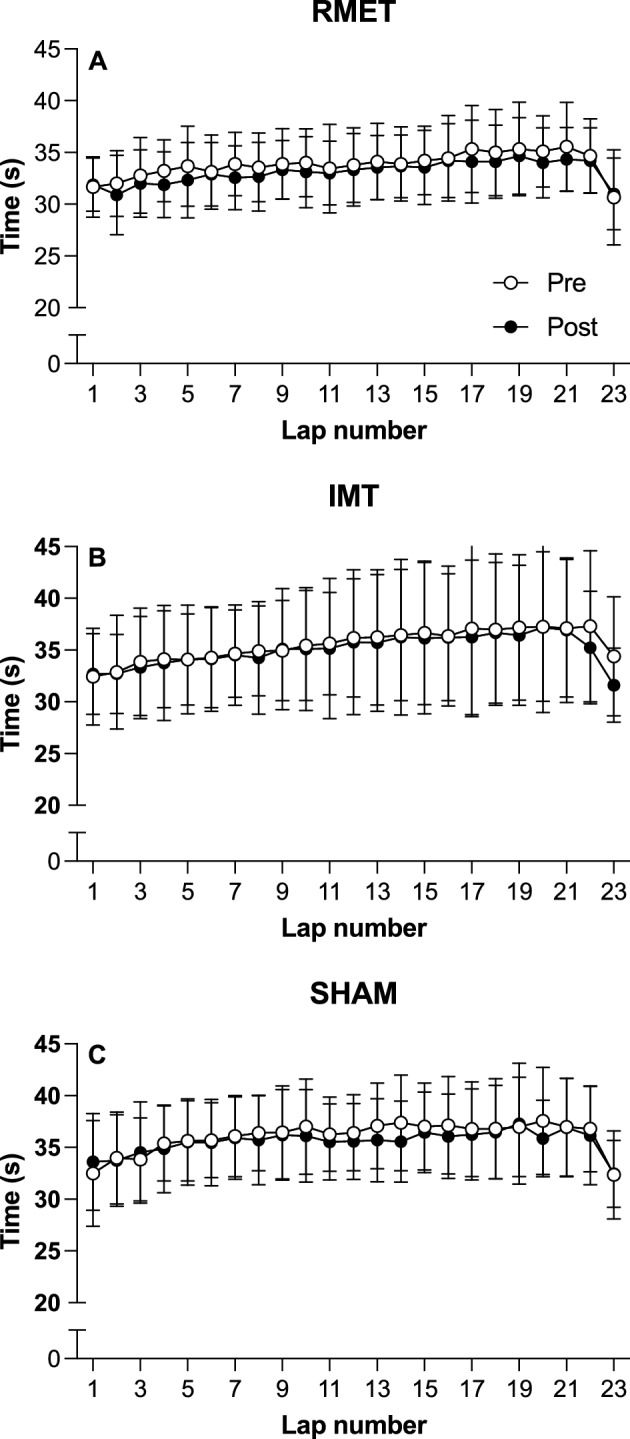
Lap-by-lap performance during the 3-km time-trial performed before (open symbols) and after (closed symbols) (**A**) respiratory muscle endurance training (RMET); (**B**) inspiratory muscle resistive training (IMT); or (**C**) SHAM training. Data are mean ± SD. There was a main effect of training (*p* = 0.026 indicating superior performance after the training period when all three groups were pooled.

3-km running time-trial performance and physiological responses are shown in Fig. 4, whereas a more detailed view of lap-by-lap performance during the 3-km TT is shown in Fig. 5. There were no differences between groups at pre-training (RMET 13.0 ± 1.3 min, IMT 13.4 ± 2.0 min, SHAM 13.8 ± 1.4 min, all pairwise comparisons *p* ≥ 0.638). All three groups were significantly faster after the training period (RMET − 1.9 ± 2.4%, Fig. 5A; IMT − 1.3 ± 2.7%, Fig. 5B; and SHAM − 1.4 ± 4.4%, Fig. 5C; *p* = 0.026 for main effect of training, Fig. 4A). There were also no differences between the groups after the training period. Average heart rate during the time-trial (group-training interaction *p* = 0.346, Fig. 4C) and blood lactate concentration immediately after the time-trial (group-training interaction *p* = 0.716, Fig. 4B) were not different between the groups either before or after the training period.

## Discussion

The main findings of the present study were that—contrary to our hypothesis—neither RMET nor IMT improved running or cycling endurance performance beyond the changes seen in the SHAM group. Similarly, respiratory muscle training did not change the amount of quadriceps fatigue measured at the end of an equal amount of cycling. Finally, all three groups elicited changes in breathing pattern which might be related to a decrease in the sensation of respiratory exertion.

### Effects of different RMT on respiratory muscle function

Both RMET and IMT improved respiratory muscle function beyond the changes seen in SHAM, confirming the efficiency of both training methods. Additionally, only the IMT group improved MIP following training, which confirms the specificity of respiratory muscle adaptation to different stimuli. Although the increase in respiratory endurance did not differ between both training groups, the fact that all participants in the RMET-group reached the 40-min time limit while less than 70% of the participants in the IMT-group reached this limit suggests a greater effect on respiratory muscle endurance after RMET, as already explored^13,33^. Indeed, a recent meta-analysis suggests that IMT is less effective than RMET in improving respiratory muscle endurance^28^, again arguing for a high specificity in training adaptations.

The increases in MVV_15_ seen after IMT and RMET were similar to those exhibited by the SHAM group. This is in agreement to what has been found in a different study, where RMET or a sprint-interval-based training also increased MVV_12_, but not more than a SHAM training group^34^. Notably, in the present study even the increase in V_T_ during the MVV_15_ test was similar between groups, which was also the case during the incremental exercise test and cycling endurance test. MVV maneuvers are less likely to show improvement following periods of RMT^28^, possibly depending on factors other than inspiratory muscle force or endurance. The fact that all three groups changed their breathing pattern during the maneuver after training supports a greater role for coordination in determining the outcome of the test, as a right balance between the deepness and frequency of the breaths is required to generate the highest possible pulmonary ventilation. Our SHAM group performed several slow, deep inhalations as part of their training, in addition to MVV_15_ maneuvers to track their progress during the training sessions, which may explain the shift towards larger tidal volumes during the test. Indeed, the above-mentioned study by Schaer et al.^34^ also used deep breathing as part of the SHAM training, which might explain the similarity in results.

### Effects of different RMT on whole-body exercise

The lack of changes in running or cycling performance is at odds with two meta-analysis suggesting enhancements in time-trial performance following RMT^4,5^, but in line with other studies which also found no ergogenic effects of RMT in the absence^38,39^ or presence of sham or control groups^40,41^. The reasons for the discrepancy are not immediately clear, as RMT has been shown to improve performance in sports ranging from cycling^11^ to running^6^ and rowing^12^.

RMT is hypothesized to improve exercise performance via an increase in leg muscle fatigue-resistance, the latter stemming from reduced or delayed activation of the respiratory muscle metaboreflex^2,21,22^. Alternatively, improvements in performance might result from a decreased perception of breathlessness/breathing exertion^7,11,12,27^ independent of the metaboreflex. While a decrease in respiratory exertion was noted during the cycling endurance test after both RMT programs, this was similar in magnitude to the effect seen in the SHAM group and therefore unlikely to justify any ergogenic effects.

Likewise, the changes in breathing pattern seen during the cycling endurance test – when ${\dot{\text{V}}}$_E_ was coached to a target level – were also seen during the MVV_15_ and incremental tests, where no target ${\dot{\text{V}}}$_E_ was given. Thus, changes in breathing pattern were robust across a variety of tests and seem to be an actual adaptation to the different training regimens, including the slow deep breaths in the SHAM group. These changes, however, also seem to be insufficient to yield ergogenic effects at whole-body level.

### Effects of different RMT on quadriceps contractility

No changes in the extent of quadriceps fatigue developed after high-intensity cycling was noted following either IMT or RMET. This was true when comparing quadriceps fatigue after equal amount of leg work had been performed (the first part of the cycling endurance test) or at exhaustion (the second part of the cycling endurance test). This is in stark opposition to the expected effects of the respiratory muscle metaboreflex, and also in contradiction with the findings from acute studies, where loading and unloading the respiratory muscles can respectively potentiate and alleviate the extent of peripheral fatigue developed during cycling^20,42^.

The present data corroborates the only two other studies performed on this topic using whole-body exercise: first, Verges et al.^41^ demonstrated that although RMT improved respiratory endurance, it did not attenuate the level of diaphragmatic fatigue developed after high-intensity cycling. More recently, Schaer et al.^23^ reported that neither traditional RMET nor a sprint-interval variation of the protocol^43^ elicited changes in quadriceps fatigue following an equal amount of high-intensity cycling. Taken together, the present data and previous studies suggest that any ergogenic effect of RMT is more likely  independent of a direct attenuation of lower limb fatigue caused by more fatigue resistant respiratory muscles. In this direction, a decrease in the perception of leg exertion was noted at the end of the first cycling bout, although it was not distinguishable from the effects of the SHAM group.

### Limitations

When interpreting the results from the present study, it must be kept in mind that during the cycling endurance tests, even though ${\dot{\text{V}}}$_E_ was coached by the experimenters to reach the same values before and after training, ${\dot{\text{V}}}$_E_ was slightly lower in the experiments performed after the training programs. Important, however, differences of this magnitude have been shown to be unimportant for the development of respiratory or quadriceps fatigue^44^. Nonetheless, as decreased respiratory muscle work increases endurance performance, this reduction in ${\dot{\text{V}}}$_E_ should have amplified the ergogenic effect of RMT, if anything. Finally, while the present investigation focused on men only, respiratory muscle training studies comparing the responses of men and women performed to date do not show a distinctively different response in women compared to men^39^. Nonetheless, given the relatively smaller lungs of women for a given body size and the higher O_2_ cost of ventilation, more trials focusing specifically on potential sex differences in training responses are needed.

Finally, while our participants were active individuals, a possible lack of familiarity with the specific performance tests could have affected our results. However, we note that the pacing profile during the 3-km time-trials was very similar between the assessments performed before and after the training period — which would not be expected if the improvements in performance were the result of familiarization with the time-trial settings alone. In the case of cycling, the objective measures of quadriceps contractility agree with the lack of performance improvement, again suggesting robustness of the findings.

## Conclusion

In conclusion, the present study showed that both RMET and IMT increased respiratory muscle endurance while only IMT improved maximal inspiratory pressure. However, neither training regimen was able to improve whole-body exercise performance during running or cycling, which was corroborated by a lack of change in quadriceps fatigue after high-intensity cycling. Therefore, the current study questions the ergogenic effect of RMT and the role of respiratory muscles in regulating the extent of peripheral fatigue developed during high-intensity exercise in healthy, active individuals.

## Data Availability

The datasets generated during and/or analyzed during the current study are available from the corresponding author on reasonable request.
